# Accuracy of MRI Pelvis in the Diagnosis of Ovarian Endometrioma: Using Histopathology as Gold Standard

**DOI:** 10.7759/cureus.20650

**Published:** 2021-12-23

**Authors:** Saad Siddiqui, Vaqar Bari

**Affiliations:** 1 Radiology, Northwest School of Medicine, Peshawar, PAK; 2 Radiology, Aga Khan University, Karachi, PAK

**Keywords:** pelvic pain, ovarian cyst., infertility, endometeriosis, magnetic resonance imaging

## Abstract

Background

Endometriosis is defined as the ectopic presence of endometrial mucosa at locations other than the uterine cavity. It results in significant morbidity and is a leading cause of infertility as well. Magnetic resonance imaging (MRI) is establishing its role in the diagnosis of endometriosis and its complications. The objective of this study was to assess the accuracy of MRI in the diagnosis of ovarian endometriosis using histopathology as the gold standard.

Materials and methods

All patients presenting with clinical complaints and suspicion of endometriosis, undergoing MRI followed by surgical resection and confirmation by histopathology, were included in the study. Data were analyzed on a predefined proforma and parameters of accuracy were calculated.

Results

A total of 170 patients were included in this study, having a mean age of 36.8 years with a standard deviation of 10.4 years. The mean parity for included subjects was 2.25, with a standard deviation of 1.3. Overall, the sensitivity of MRI was 86.7% and the specificity was 81.9%. The positive predictive value (PPV) of MRI was 83.3%, while the negative predictive value (NPV) was 81.9%. Overall, the diagnostic accuracy of MRI was 84.7%.

Conclusion

The accuracy of MRI in the diagnosis of endometriosis was found to be acceptable and comparable to most of the worldwide published literature. The routine use of MRI for diagnosis and preoperative planning is justified by the results of this study.

## Introduction

Endometriosis is defined as the ectopic presence of endometrial mucosa at locations other than the uterine cavity. It usually results in the formation of a cystic structure called an endometrioma. Although it can be found anywhere in the body, the ovary is the most frequent site for endometrioma formation. The ectopic tissue is hormonally responsive and may undergo bleeding, inflammation, fibrosis, and eventually adhesion formation, often resulting in infertility. Acute exacerbations of endometriosis are often secondary to chemical peritonitis due to leakage of old blood from cysts [1].

The usual signs and symptoms of endometriosis are dysmenorrhea, menorrhagia, pelvic or lower abdominal pain, dyspareunia, bloating/gastrointestinal symptoms, and dysuria. Endometriosis frequently results in infertility or subfertility and may be incidentally diagnosed in patients undergoing evaluation for infertility. Complications of endometriosis include malignant transformation, endometrioma rupture, and secondary infection of endometrioma [1].

Laparoscopy remains the gold standard in diagnosis of endometriosis; however, imaging usually precedes invasive methods of diagnosis and treatment in patients with pelvic pain, subfertility or adnexal mass. Transvaginal sonography is usually the first modality employed; however, it has limitations in terms of low reported sensitivity (45%) and inability to identify secondary complications like adhesion formation and peritoneal endometriotic implants. Magnetic resonance imaging (MRI) is therefore establishing its role in the diagnosis of endometriosis and its complications. MRI is also particularly useful in patients who are virgo intacta and hence not suitable candidates for transvaginal sonography. MRI has the additional advantage of being able to characterize tissue types as fat, blood, etc. MRI can also detect any significant associated findings like adhesion formation, hemorrhage, and secondary implants [2-6].

The objective of this study was to determine the diagnostic accuracy of MRI pelvis in the diagnosis of ovarian endometrioma in patients presenting with recurrent pelvic pain, dysmenorrhea, dyspareunia, and primary or secondary infertility at a tertiary care hospital, using histopathology as the gold standard.

## Materials and methods

This is a cross-sectional study performed at the radiology department of a tertiary care health facility for a period of one year. Sampling was done by non-probability, consecutive sampling. The sample size was calculated using a sample size calculator for sensitivity and specificity studies by Lin Naing. From published literature, the reported sensitivity of MRI in the diagnosis of endometriosis is 94% and the specificity is 97%. The expected prevalence in Pakistan was reported as 24.8%. Keeping the desired precision at 0.06 for sensitivity and 0.03 for specificity, respectively, a confidence level of 95%, the required sample size was calculated as 170 [7].

All female patients aged 14 to 65 years, referred for MRI pelvis for evaluation of endometriosis, including both pre-menopausal and post-menopausal patients, were made part of the study. Patients having a prior history of biopsy proven endometriosis and patients for whom the result of final histopathology was not available were excluded.

All examinations were performed on a 1.5 Tesla MRI scanner by Siemens Healthcare, Germany, using a torso phased-array coil for the abdominal scan and a pelvic array coil for the pelvic scan. The sequences acquired included axial T1-weighted, axial, coronal and sagittal T2-weighted, axial T1-weighted with fat suppression, diffusion-weighted imaging (DWI) and apparent diffusion coefficient (ADC) maps. Gadolinium contrast injection was administered at a dose of 0.2 mmol/kg and post-contrast T1-weighted image acquisition was performed in the axial, coronal and sagittal planes with fat suppression. Slice thickness was set at 5 mm with a gap of 1 mm except for volumetric images of post contrast sagittal images. All acquisitions were archived and processed on Picture Archiving and Communication Systems (PACS). Reporting of images was performed on a 5 mega pixel diagnostic console by a radiologist.

Patients with ovarian lesions showing MRI features of endometrioma (i.e., hyperintense on T1 and T1 fat suppressed images, hypointense on T2-weighted images, and following signal characteristics of blood products on DWI and ADC map) were labeled as MRI positive. Patients with ovarian lesions that did not follow the imaging characteristics of an endometrioma were labeled as MRI negative.

Similarly, patients with resected ovarian lesions that had two out of three features: the presence of endometrial glands, endometrial stroma, or hemorrhage on microscopy were labeled as "histopathology positive." All other patients with resected ovarian lesions that did not fulfill two out of the three aforementioned criteria were labeled as "histopathology negative."

Patients who were both MRI positive and histopathology positive were labeled as "True Positives." Patients who were MRI positive but histopathology negative were labeled as "False Positives." Patients who were both MRI negative as well as histopathology negative were grouped as true negatives, while patients who were MRI negative but histopathology positive were grouped as false negatives. Sensitivity, specificity, positive predictive value (PPV), negative predictive value (NPV), and diagnostic accuracy were then calculated (Table 1).

**Table 1:** 2 x 2 table for calculating the sensitivity, specificity, positive predictive value, negative predictive value of MRI using Histopathology as gold standard.

		Histopathology		Total
		Endometriosis present	Endometriosis absent	
MRI	Endometriosis present	85	13	98
	Endometriosis absent	13	59	72
Total		98	72	170

Formal approval was obtained from the ethical review committee of the institution prior to the initiation of the study. The data were collected from patients who were already referred for MRI examination with clinical suspicion of endometriosis and no further imaging was done which could have helped in results of this study. Therefore, no potential radiation exposure or additional financial constraint was caused to patients. The participant had the full right to refuse participation without any consequences. The complete privacy and confidentiality of participants was ensured.

## Results

**Figure 1:**
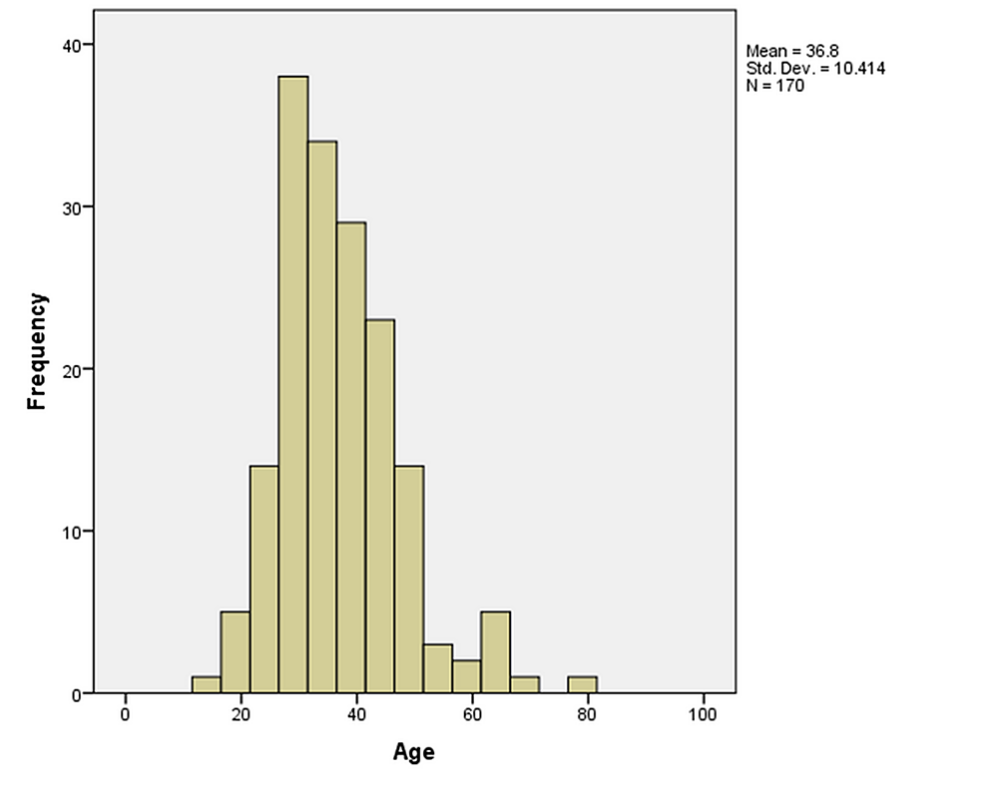
Age wise distribution of cases.

**Figure 2:**
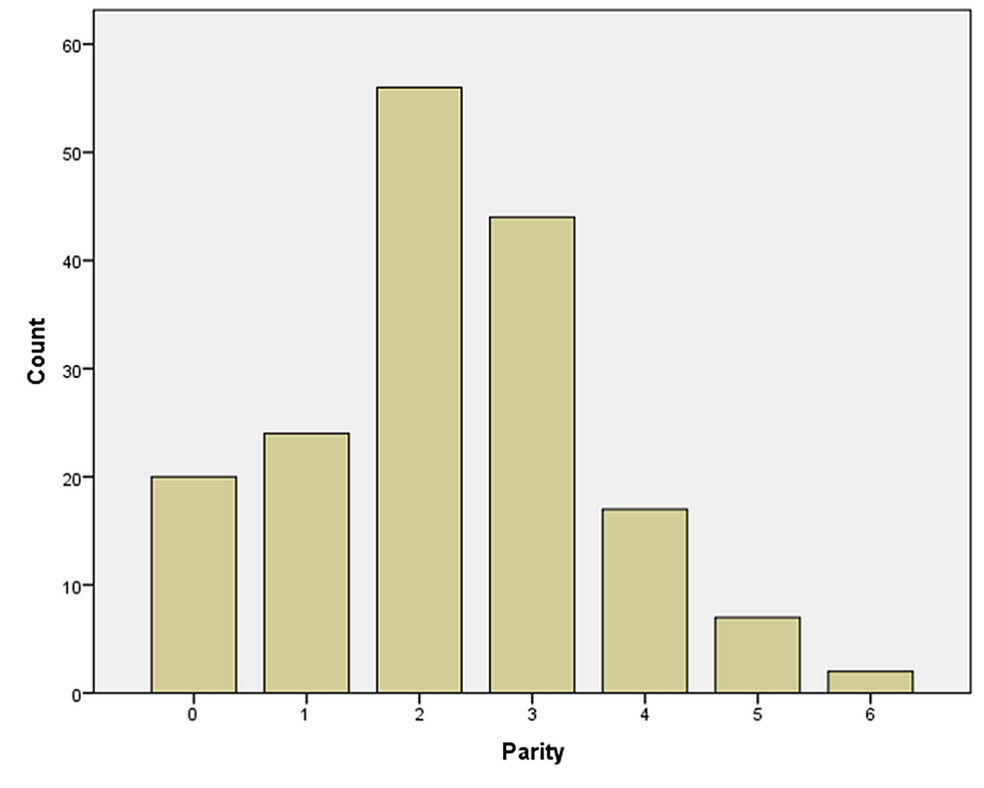
Distribution of cases according to parity.

A total of 170 patients were included in this study. The mean age of subjects included in the study was 36.8 years, with a standard deviation of 10.4 years. The age range was from 14 years old to 80 years old. The histogram shows the distribution of study subjects according to age (Figure 1). The mean parity for included subjects was 2.25, with a standard deviation of 1.3. Parity ranged from 0 to 6 (Figure 2). Similarly, 88.2% of the participants were premenopausal, while 11.2% were postmenopausal.

Overall, MRI diagnosed endometriosis in 98 out of 170 cases. Among these, 85 out of 98 (86.7%) patients were true positives. There were 13 out of 98 positive cases that were false positives (18.1%). The MRI diagnosis classified 72 out of 170 patients as negative. Among these 59 (81.9%) were true negative, whereas 13 (13.3%) were false negative (Table 1).

The overall sensitivity of MRI was found to be 86.7%, and the specificity was 81.9%. The positive predictive value of MRI was 83.3%, while the negative predictive value was found to be 81.9% (Table 2). The diagnostic accuracy of MRI was 84.7%. In order to control potential biases, further stratification for age (Table 2) and parity (Table 3) was also performed.

**Table 2:** Parameters of accuracy stratified according to age.

Category	No of cases (n)	Sensitivity (%)	Specificity (%)	PPV (%)	NPV (%)
Overall	170	86.7	81.9	83.3	81.9
<30	43	96.4	80.0	90.0	92.3
30-39	65	88.2	93.5	93.7	87.8
40-49	47	82.8	77.8	85.7	73.7
>50	15	81.2	76.7	84.6	74.1

**Table 3:** Parameters of Accuracy stratified according to parity.

Category	No of cases (n)	Sensitivity (%)	Specificity (%)	PPV (%)	NPV (%)
Nulliparous	20	97.3	81.0	83.5	90.0
1–2	80	87.5	75.0	84.0	80.0
3–4	61	82.9	80.8	85.3	77.8
>5	9	66.7	97.0	98.0	85.7

## Discussion

Endometriosis is a major cause of morbidity worldwide. Besides causing a significant drain of resources, it also results in debilitating physical pain. As well as being a leading cause of infertility/subfertility, it is also a major cause of psychological trauma for women who are unable to start or complete their families [8-10].

It is primarily a disease of the young population, as is evident from our results, where the mean age was found to be 36.8 years with a standard deviation of 10.4 years. Being a major cause of morbidity in this young age group, it also carries a significant economic burden in two ways. First, as a result of direct costs incurred in treatment of endometriosis and its complications; and second, as a result of loss of productive manpower, especially in developed countries [8,10,11].

The mean parity for included subjects was 2.25, with a standard deviation of 1.3. This is also less than the average figure of 3.2 for our country, as available from published literature by the United Nations. The reason for this lower value than the national average could be due to infertility or subfertility caused by endometriosis [5,6].

In Pakistan, the prevalence of ovarian cancer is reported to be in the range of 5.0 to 8.8 per 100,000. A relatively high figure has also been reported as the incidence of endometriosis in Pakistan. Considering the high association of ovarian cancer and endometriosis, it is possible that endometriosis is the underlying cause in many of these cases. Further work in this regard is needed to establish exact figures [7-13].

The overall sensitivity of MRI was found to be 86.7%, and the specificity was 81.9%. These results were in closer agreement with studies from the USA and much lower than figures reported from Italy. Possible reasons for this could be the selection criteria of patients who are referred for MRI or a difference in training and experience of the interpreting radiologist. Even factors like self-paid versus state-sponsored healthcare may play an important role in determining patients who undergo evaluation using MRI, as it is an expensive modality in most countries with significant waiting time for appointments [14,15].

Most of the false positive cases turned out to be teratoma on histopathology. They can have quite a lot of overlapping features on MRI as both may contain heterogeneous internal contents and appear hyperintense on T1-weighted sequences secondary to blood products in an endometrioma and fat content in a teratoma. Although fat-suppressed sequences are routinely acquired as part of the protocol in the department where this study was conducted, some lesions were still misinterpreted as endometrioma and turned out to be teratoma on histopathology [15,16].

Another common mimicker of endometriosis identified during data collection was adenomyosis. This was also cause of significant morbidity in relatively young patients just like endometriosis, however, a detailed discussion of adenomyosis beyond scope of this study and further work from our region would be recommended [17,18].

While performing a literature search for this work, studies were identified that mention a significantly high risk of endometrial cancer in patients with endometriosis. Although the relationship between endometriosis and the development of ovarian cancer is well established now, its association with endometrial carcinoma remains yet to be completely established. However, as MRI is currently the first line of modality for staging of endometrial carcinoma, it would further enhance the role of MRI in the diagnosis of endometriosis and its complications [19-21].

Transvaginal sonography is also reported to be a reasonable alternative to MRI in the diagnosis of endometriosis, including the deep-infiltrating type. It can provide a cost-effective, relatively easy-to-access and quick alternative for the evaluation of endometriosis. However, as per the opinion of the authors of this work, its operator dependability as well as its inability to adequately assess peritoneal and bowel wall deposits would still make MRI a superior alternative in routine practice. Deep-seated endometriosis has also been reported to involve the appendix, and some authors have even proposed appendectomy in patients undergoing surgery for deep infiltrating endometriosis. In such cases, evaluation by transvaginal ultrasound would be insufficient as it is likely to miss appendicular deposits [22].

Additional sequences, although part of routine protocol in our department, may not be routinely performed as per protocol in other departments. Prime among these sequences is DWI. Some authors found diffusion-weighted imaging to be especially helpful in the diagnosis of deep pelvic endometrial deposits, which showed consistently low apparent diffusion coefficient values [23]. Additional techniques like Cine MRI are also establishing their role. However, at the moment, their applicability is limited and they are mostly used for research purposes [23].

Hence, there are a couple of indirect features on MRI which may suggest the presence of adhesions in patients with endometriosis and hence enable surgeons to plan extensively pre-operatively for resection and conservation rather than make on-table decisions. MRI allows identification of the location, size, and number of endometriomas or implants. Low-signal-intensity stranding that obscures organ interfaces is an important finding for the identification of adhesions. Posterior displacement of the uterus, "kissing" ovaries, angulated bowel loops, an elevated posterior vaginal fornix, loculated fluid collections, hydrosalpinx, and hematosalpinx are additional findings that indicate adhesions. Multiphase and multisection MR imaging with kinetic display may allow prediction of pelvic adhesions. In a study, organ movement was analyzed with a half-Fourier acquisition single-shot fast spin-echo sequence. It provided a sensitivity of 72.5% and a specificity of 87.4% for the diagnosis of adhesions. Hematosalpinx was also a commonly associated finding in our study, and almost all of these cases had involvement of fallopian tubes by endometrial deposits.

As evidenced by published work presentations [24], endometriosis diagnosis is significantly delayed in developed European countries. Considering the socioeconomic factors of Pakistan, presentation may be even more delayed, and that would mean more complicated endometriosis. This indirectly further enhances the role of MRI in diagnosis and preoperative planning in cases of endometriosis [7,24].

A limitation of this study was that it only assessed ovarian endometriomas. The reported involvement of the ovaries in endometriosis is around 67%. The authors would recommend further research to establish the accuracy of MRI in diagnosing endometriomas at sites other than the ovaries [25].

## Conclusions

MRI has an established role in the diagnosis and preoperative staging of endometriosis. This study validates the role of MRI, even in developing nations. The results of this study justify the routine use of MRI in the diagnosis and preoperative staging of endometriosis.
